# Estimation of Tensile Modulus of a Thermoplastic Material from Dynamic Mechanical Analysis: Application to Polyamide 66

**DOI:** 10.3390/polym14061210

**Published:** 2022-03-17

**Authors:** Albert Serra-Aguila, Josep Maria Puigoriol-Forcada, Guillermo Reyes, Joaquin Menacho

**Affiliations:** 1 Passive Safety Department, Applus + IDIADA HQ, Santa Oliva, L’Albornar, P.O. Box 20, 43710 Tarragona, Spain; albert.serra@idiada.com; 2 IQS School of Engineering, Universitat Ramon Llull, Via Augusta 390, 08017 Barcelona, Spain; josep.puigoriol@iqs.url.edu (J.M.P.-F.); guillermo.reyes@iqs.url.edu (G.R.)

**Keywords:** polymer characterization, mechanical properties, viscoelasticity, DMA

## Abstract

The mechanical properties of thermoplastic materials depend on temperature and strain rate. This study examined the development of a procedure to predict tensile moduli at different strain rates and temperatures, using experimental data from three-point-bending dynamic mechanical analysis (DMA). The method integrated different classical concepts of rheology to establish a closed formulation that will allow researchers save an important amount of time. Furthermore, it implied a significant decrease in the number of tests when compared to the commonly used procedure with a universal testing machine (UTM). The method was validated by means of a prediction of tensile moduli of polyamide PA66 in the linear elastic range, over a temperature range that included the glass-transition temperature. The method was applicable to thermo-rheologically simple materials under the hypotheses of isotropy, homogeneity, small deformations, and linear viscoelasticity. This method could be applicable to other thermoplastic materials, although it must be tested using these other materials to determine to what extent it can be applied reliably.

## 1. Introduction

There are a variety of engineering applications involving viscoelastic properties of materials, ranging from the more classic structural applications [1,2], through nanocomposites and biopolymers [3], to biomechanical models [4,5] and biomedical engineering [6,7]. There is also a variety of mathematical models to fit the performance of materials that have viscoelastic behavior [8,9]. In spite of this variety of models, such as quasilinear models [10,11], fractional models [12,13], and other nonlinear models [14,15], linear models in the form of a Prony series are the simplest and therefore the most widely used in industrial practice [8,16]. In order to create mathematical models that allow for good calculations, the identification of the parameters of viscoelastic material models is a permanent issue in engineering research [17,18]. The limits of an accurate identification imply limitations to the applications in many engineering fields [5,7,19]. This task is more difficult, since these models must take into account the variation in the behavior of materials at different frequencies and temperatures [2,6,20]. Although dynamic mechanical analysis (DMA) is a well-established option for obtaining material-response data over a broad spectrum of frequencies and temperatures [21], new methods are currently being tested and developed to identify the material parameters: for applications at the micro- and nanoscale by atomic force microscopy [22,23,24] or optical tweezer [25]; for biological materials (challenging because of their intrinsic softness and labile nature) [26]; and for noncontact methods such as magnetic mechanical testing for biomaterials [27] or ultrasonic DMA for nondestructive inspection [28]. On the other hand, a number of studies have examined simpler methods to be able to estimate the properties of materials, reducing the experimental cost and complexity [18,29,30].

The use of a universal testing machine (UTM) to characterize a material is widespread. In industry, it is common to have the tensile modulus at room temperature and at a low strain rate (given by the supplier), but data on other temperatures and strain rates are of interest as well. However, using a UTM to determine viscoelastic properties requires, in general, a large number of tests. So, it takes a long time for a complete characterization. Therefore, the progress and challenge of this field is to estimate a large amount of information in a shorter time, by using only dynamic mechanical analysis (DMA) [18,21]. After the experimentation with DMA, if the information is treated through a series of mathematical models, it is possible to obtain the same information as in the use of a UTM, without the necessity of using this device. This procedure allows researchers to save time and money.

To avoid the time-consuming experimentation in UTM tests, the present article deals with experimental data from DMA tests to obtain tensile moduli of thermoplastic materials. Three-point-bending DMA experimental data were measured at certain values of amplitude, frequency, and temperature. With these input data, a mathematical model was created and its parameters were found, in order to predict the tensile moduli in the linear elastic zone for a range of temperatures and strain rates.

Consequently, the objective of this work was to predict the tensile modulus of polyamide PA66 as a function of temperature and strain rate from three-point-bending DMA data without using a UTM. The validation of the results was carried out by comparing them with experimental data.

Three hypotheses to be validated were assumed:

1. The relationships between the flexural modulus and frequency, and also between tensile modulus and strain rate, follow a logarithmic tendency;
2. There is an equivalence between the strain rate of a tensile test and the frequency and amplitude of the sinusoidal strain from the flexural tests;
3. There is a factor that permits a relationship between the tensile modulus and flexural modulus. This factor is not dependent on strain rate nor on temperature.

From these assumptions, the mathematical model was established. Then, it was applied to a polyamide as an example. At the end of the work, the hypotheses will be verified when contrasted with the experimentation.

The procedure began with the DMA experimental data (storage and loss moduli) over input parameters (amplitude, frequency, and temperature). Once these data were measured, with the use of the proposed mathematical model, tensile moduli were estimated at different temperatures and frequencies. Finally, these estimated values were compared to tensile tests in order to validate the material model.

## 2. Materials and Methods

**Figure 1:**
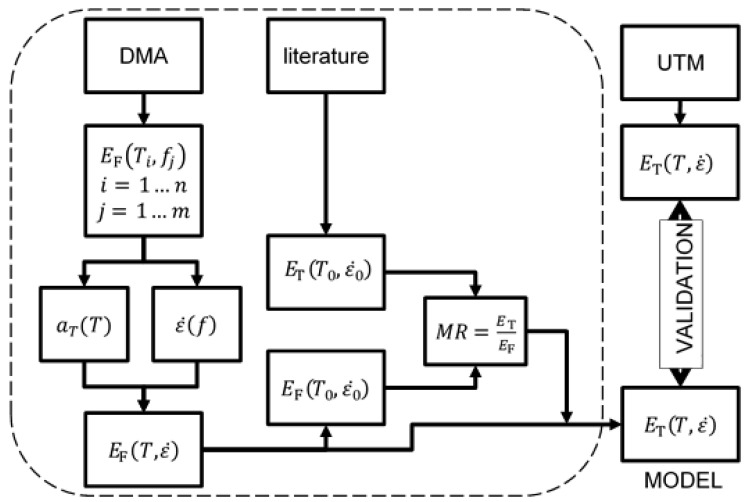
Diagram of the “tensile loop”.

The diagram in Figure 1 shows the concept of the present work. Starting from DMA data: on one hand, when comparing the flexural modulus (*E*_F_) for different temperatures and frequencies, it was possible to state the factors for interconverting between temperatures and frequencies $\left(a_{T}\right)$; on the other hand, a relationship between frequency and strain rate was determined ($\dot{\varepsilon }\left(f\right)$). As a result, the flexural modulus was calculated as a function of the temperature and strain rate. Assuming that the tensile modulus of the material was known from the literature, just for a single reference temperature and strain rate, the flexural modulus could then be calculated in the same reference conditions. This allowed us to state the relationship (*MR*) between the tensile and flexural moduli (third hypothesis of this work). As a result, a model could be built to predict the tensile modulus for every temperature and strain rate (in a certain range). Finally, it was possible to define a loop check (tensile loop) by using data from the UTM to validate the model.

### 2.1. Mathematical Material Model

In order to state the mathematical model of a viscoelastic material, two different types of models were checked in the literature:

- Models depending on the strain rate: in which the mechanical property under study was defined as a function of the strain rate;
- Models depending on the temperature: in which the mechanical property under study was defined as a function of temperature.

#### 2.1.1. Strain Rate Dependence

In polymers and composites, the modulus and the strength increase with an increase in the strain rate [16,31]. The relationship between these mechanical properties (modulus or strength) follow a logarithmic relationship as a function of the strain rate (first hypothesis of this work). One of the most well-known models that describes mechanical properties as a function of strain rate is the Johnson–Cook model [32,33].

The Johnson–Cook model is a mechanical model that was developed to describe the plastic behavior that a material suffers under impact conditions. The behavior of materials subjected to high strains, high temperatures, and high strain rates is reproduced. This model is defined using parameters measured in experimental procedures at different strain rates and temperatures. The yield stress is defined as [32]:

(1) $$\sigma =\left(\sigma_{0}+JC_{1}\cdot \;\varepsilon_{p}{}^{JC_{2}}\right)\cdot \left(1+JC_{3}\cdot \ln{\dot{\varepsilon }}_{p}^{*}\right)\cdot \left(1-T_{H}{}^{JC_{4}}\right)$$

where σ is the stress, $\sigma_{0}$ the yield stress at the reference temperature and at a low strain rate; $\varepsilon_{p}$ the effective plastic strain; ${\dot{\varepsilon }}_{p}^{*}$ the effective plastic strain rate, normalized with the reference strain rate $\dot{\varepsilon_{0}}=1\;s^{-1}$; $JC_{i}\;\left(i=1\ldots 4\right)$ are four coefficients of the Johnson–Cook model; and *T*_H_ is a dimensionless coefficient. This last coefficient is defined as [32,33]:

(2) $$T_{H}=\frac{T-T_{\mathrm{ref}}}{T_{m}-T_{\mathrm{ref}}}$$

where *T* is the temperature, *T*_ref_ is the reference temperature (the ambient temperature, for instance), and *T*_m_ is the melting temperature of the material.

Although the Johnson–Cook model is related to the development of plastic strain, and the analysis of this work was related to thermoplastic materials within the linear viscoelastic region, this model was considered important in this study due to the logarithmic relationship between the modulus or strength and the strain rate.

Relying on this model, the present work proposed the first hypothesis mentioned above: the flexural modulus (or the tensile one) has, in general, a relationship with the strain rate that can be expressed by means of a logarithmic relationship of the form:

(3) $$E_{F}\left(\dot{\varepsilon }\right)=E_{F}\left({\dot{\varepsilon }}_{\mathrm{ref}}\right)+SE_{F}\cdot \ln\dot{\varepsilon }$$

where $\dot{\varepsilon }$ is the strain rate, $E_{F}\left(\dot{\varepsilon }\right)$ is the flexural modulus of the material for a strain rate $\dot{\varepsilon }$, and the reference strain rate ${\dot{\varepsilon }}_{\mathrm{ref}}=1\;\mathrm{mm}/\min$. $SE_{F}$ is the slope of the linearized expression, and can be calculated by fitting a line to the experimental data.

As will be seen below, a completely analogous expression was proposed for the tensile modulus.

The units for strain rate will always be mm/min in this work, in order to compare the different strain rates measured in tensile tests with the UTM results. This input parameter (strain rate) for these tests is given in mm/min as well.

#### 2.1.2. Temperature Dependence

It is well known that in polymers and composites, the modulus decreases with an increase in temperature [16,34]. The relationships between mechanical properties as a function of temperature have been widely studied. Several constitutive properties (such as relaxation modulus or creep compliance) depend on temperature and time. The time–temperature superposition principle, as found in the literature [16,35,36], states that there is a certain equivalence between a variation in temperature and the time scale in which the processes of relaxation, creep, or deformation take place. For example, relaxation time varies in a certain (inverse) proportion with temperature. This means that the curves of a certain constitutive property with respect to time (or frequency) obtained at a higher temperature can be superimposed on those obtained at a lower temperature by applying a suitable change of scale to the time axis. Since the temporal (or frequency) scales used in this type of experiment are logarithmic, the change in time scale corresponds to a shift in the (logarithmic) time axis. The materials in which this principle of time–temperature superposition applies are called thermo-rheologically simple. In the present study, in order to deal with the influence of the temperature, a shift factor was used, *a_T_*. The shift factor could be calculated with a shifting procedure.

Knowing how this shift factor affects the temperature is of great interest. The shift factor is defined as [16]:

(4) $$a_{T}=\frac{t_{T}}{t_{T_{\mathrm{ref}}}}$$

where *a_T_* is the shift factor, *t_T_* is the time to reach a certain value in a mechanical property (relaxation modulus or creep compliance) at temperature *T*, and *t_T_*_ref_ is the time to reach a certain value in a mechanical property at reference temperature *T*_ref_. This factor accounts for the time–temperature superposition of the relaxation modulus (or creep compliance), so that *t* units of time at temperature *T* are equivalent to $t/a_{T}$ units at temperature $T_{\mathrm{ref}}$.

The shift factor also applies to constitutive functions expressed in the frequency domain. For the complex modulus $G^{*}=G^{\prime }+j\cdot G^{\prime \prime }$, it leads to [16]:

(5) $$G^{*}\left(f,T\right)=G^{*}\left(a_{T}\cdot f,T_{\mathrm{ref}}\right)$$

Various empirical equations have been proposed to determine the temperature-dependent behavior of the shift. The Williams–Landel–Ferry (WLF) equation is useful when the material has a temperature above the glass transition *T_g_*, as well as in the *T_g_* region. This equation uses the following expression [16,34]:

(6) $$\log a_{T}=\frac{-C_{1}\left(T-T_{\mathrm{ref}}\right)}{C_{2}+\left(T-T_{\mathrm{ref}}\right)}$$

where *T*_ref_ is the reference temperature, and *C*_1_ and *C*_2_ the coefficients of the WLF equation. The values of the constants initially were thought to be universal constants, taking values of 17.44 and 51.6, respectively, when *T*_ref_ was considered *T_g_*. Today, they are instead considered as parameters to curve-fit in each case [16].

When the temperature is below *T_g_*, the relationship between the shift factor and temperature is given by the Arrhenius equation [37], which is defined using the following expression:

(7) $$\log a_{T}=\frac{E_{a}}{2.303\cdot \;R}\cdot \left(\frac{1}{T}-\frac{1}{T_{\mathrm{ref}}}\right)$$

where *E_a_* is the activation energy, *T* is the (absolute) temperature, and *R* the thermodynamic constant.

When working at different temperatures with WLF or Arrhenius shift functions, the number of temperatures to be analyzed in order to achieve a reliable adjustment of the shift factor over temperature depends on how much time the experiment lasts. In most cases, 4 or 5 temperatures can be enough [38]. The coefficients for the equations were found through the least-squares error method, after the equations were linearized [39]. 

In order to determine the shift coefficient of a material, this work began with the curves of the storage and loss moduli over a range of frequencies, provided by DMA tests at different temperatures. The curves given by these tests were shifted horizontally until they overlapped (at least in a certain range). The values of these shifts were then adjusted to the WLF or Arrhenius models.

However, according to what was previously exposed, two considerations were taken into account:

(a) In order to calculate the coefficients of shift functions, it was necessary to consider the change in behavior of the material when the glass-transition temperature was exceeded. Thus, either one temperature range or two temperature subranges needed to be used, depending on the case. The WLF equation presented a discontinuity when the test temperature was a certain temperature below *T*_ref_: when $T_{\mathrm{ref}}-T=C_{2}$. However, it was useful above *T*_ref_.

The method of finding the coefficients of the equation was through linearization of Equation (6), resulting in:

(8) $$\underset{y}{\underbrace{\frac{1}{\log a_{T}}}}=-\frac{C_{2}}{C_{1}}\cdot \underset{x}{\underbrace{\frac{1}{T-T_{\mathrm{ref}}}}}-\frac{1}{C_{1}}$$

On the other hand, the Arrhenius equation presented the energy activation, the factor of which was nonsense in temperatures above *T_g_*. However, it could be used for the subrange $T\leq T_{g}$. In this case, to find the coefficients, the linearization of (7) showed the form:

(9) $$\underset{y}{\underbrace{\log a_{T}}}=\frac{E_{a}}{2.303\cdot R}\cdot \underset{x}{\underbrace{\frac{1}{T}}}-\frac{E_{a}}{2.303\cdot R}\cdot \frac{1}{T_{\mathrm{ref}}}$$

(b) In the case in which there were two subranges, *T*_ref_ was required to be the same for every subrange in order to work within the entire temperature range indistinctly. In this way, the continuity of the values of the shift coefficient was preserved: the last temperature for the first subrange coincided with the first temperature for the second subrange; thus, the shift factor also had to coincide. Otherwise, there would be a discontinuity in the shift factor over temperature.

### 2.2. Moduli Ratio

The DMA tests carried out in this study were of a three-point-bending nature. They allowed us to determine the flexural modulus of the material. However, the goal of this work was to state the tensile modulus of the material. Therefore, it was necessary to establish a relationship between both modules. This relationship constituted the third hypothesis of this work. The proposed relationship was a simple proportion that remained constant for the considered range of temperatures and deformation rates. Hence, the moduli ratio (*MR*) was defined as the ratio of tensile modulus *E_T_* to flexural modulus *E*_F_*:*

(10) $$MR=\frac{E_{T}}{E_{F}}$$

By definition, this moduli ratio was valid for tensile and flexural moduli at the same temperature and at the same strain rate.

This ratio was considered to be independent of the temperature and strain rate (third hypothesis of this work). It meant that this parameter was assumed to be constant in the ranges of temperature and strain rate under study. With this assumption, at the end, a comparison between flexural values and experimental tensile data was always necessary. If the differences were enough low, the assumption of the existence of this constant value along the ranges of the variables under study could be considered correct.

Moreover, if the moduli ratio is applied to (3), one finds:

(11) $$E_{T}\left(\dot{\varepsilon }\right)=E_{T}\left({\dot{\varepsilon }}_{\mathrm{ref}}\right)+\underset{SE_{T}}{\underbrace{MR\cdot SE_{F}}}\cdot \ln\dot{\varepsilon }$$

So, a logarithmic line related the tensile modulus to the strain rate, and the slope of such line was that of the flexural times the moduli ratio.

### 2.3. Mathematical Model for Tensile Loop Validation

By combining the ideas outlined in the previous sections, a mathematical model for the tensile module could be constructed. That mathematical expression allowed us to calculate the tensile modulus as a function of the temperature and strain rate. This expression had two parts. One part represented the variation of tensile modulus as a function of temperature, and the other part as a function of the strain rate. The combination of both parts was the resultant tensile modulus:

(12) $$E_{T}\left(T,\dot{\varepsilon }\right)=\underset{E_{T}\left(T,{\dot{\varepsilon }}_{\mathrm{ref}}\right)}{\underbrace{MR\cdot E_{F}\left(T,{\dot{\varepsilon }}_{\mathrm{ref}}\right)}}+MR\cdot SE_{F}\cdot \ln\dot{\varepsilon }$$

The tensile modulus at a given temperature can be found from the tensile modulus at a reference temperature. Therefore, the tensile modulus presents a logarithmic adjustment as a function of the strain rate, in which the independent term is the tensile modulus at a reference temperature.

For the mathematical model of a tensile loop, the tensile modulus can be determined as a function of temperature and strain rate, as shown in (12). 

### 2.4. Material for Experiments

The experimental work was carried out with polyamide PA66 with no glass fiber. It was a lubricated and stabilized polyamide 66. It was a nylon resin with good mechanical properties, good high temperature performance, and chemical resistance. It is used in demanding applications such as in electrical and electronic devices, the automotive industry, furniture and domestic appliances, sporting goods, and packaging applications. The material used in this work was delivered by DuPont^TM^ under the tradename ZYTEL^®^ 103HSL NC010.

## 3. Experimental Application of the Method

The purpose of the method was to be able to predict the tensile modulus of a thermoplastic material (PA66) as a function of the strain rate and temperature from DMA data. 

In order to apply this method, a series of steps were followed:

1. DMA experiments (sweeps);
2. Determination of the shift factor;
3. Moduli ratio;
4. Material model: temperature- and strain-rate-dependent;
5. Validation.

### 3.1. DMA Experiments

Several amplitude, temperature, and frequency sweeps were carried out by DMA [21]. The experiments were three-point-bending tests, carried out under the ASTM D5023-07 standard. With these sweeps, storage and loss moduli were measured over these three mentioned variables.

#### 3.1.1. Amplitude Sweep

**Figure 2:**
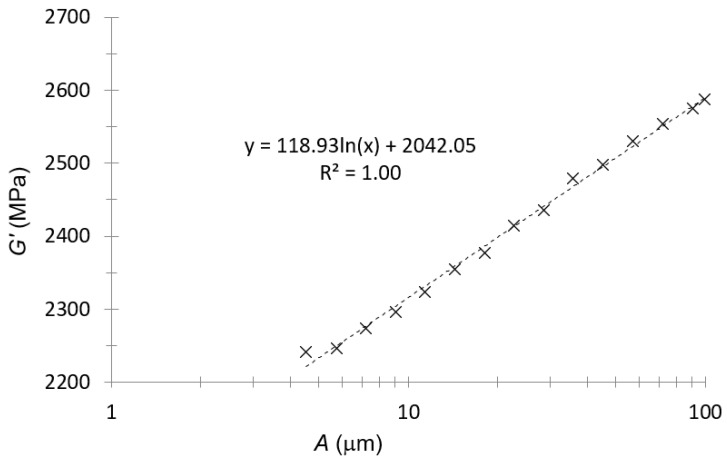
Storage modulus–amplitude plot for PA66.

The amplitude sweep was the first experimental test with DMA. This test was performed at 30 °C and at 1 Hz. The measured storage modulus $G^{\prime }\left(=E_{F}\right)$ for PA66 is presented in Figure 2.

For PA66, the amplitude to perform subsequent tests was 30 μm. This amplitude, considering the data in Figure 2, was located within the linear viscoelastic range.

**Figure 3:**
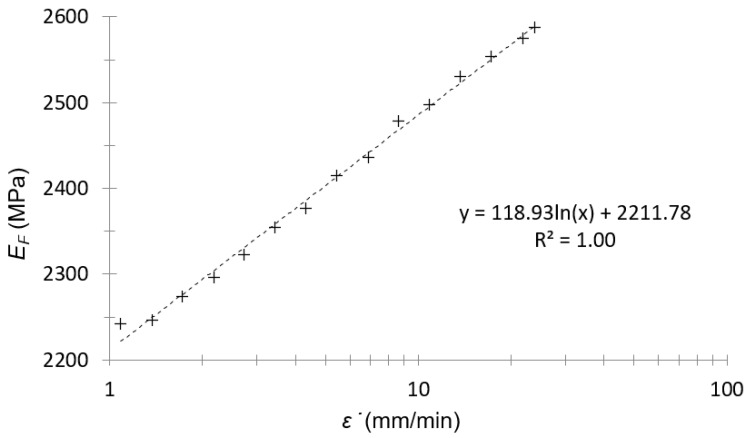
Flexural modulus–strain rate plot of amplitude sweep for PA66.

The plot in Figure 3 was only to observe if the tendency between the flexural modulus and strain rate was the expected one (a logarithmic tendency). Since the strain in the DMA tests was sinusoidal, the average strain rate was taken as the length traveled in a complete oscillation divided by a period; i.e. four times the amplitude times the frequency (second hypothesis in this work):

(13) $$\dot{\varepsilon }=4\cdot A\cdot f$$

As can be seen in Figure 3, the logarithmic tendency was confirmed between the flexural modulus and the strain rate for this material (first hypothesis of this work).

#### 3.1.2. Temperature Sweep

**Figure 4:**
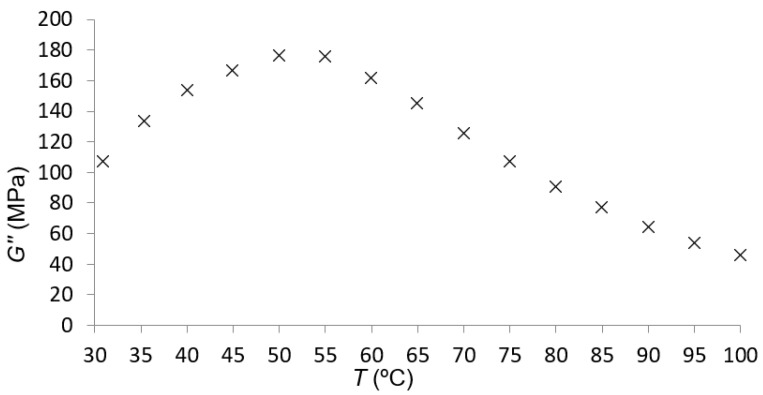
Loss modulus–temperature plot for PA66.

The temperature sweep was the second experimental test with DMA. This test was performed at 30 μm of amplitude and 1 Hz of frequency. The loss modulus for PA66 in these conditions is presented in Figure 4.

This second test was mostly used to observe if there was a maximum for the loss modulus at a specific temperature in the given conditions of amplitude and frequency.

According to the data in Figure 4, for the material under study, there was a maximum of the loss modulus at approximately 50 °C. This indicated that the subrange change, explained in Section 2.1.2, had to be considered at this temperature.

#### 3.1.3. Frequency Sweep

The frequency sweep was the third and last experimental test with DMA. This test was performed at 30 μm of amplitude at different temperatures, from 30 to 100 °C, with an increment of 10 °C between the sweeps.

**Figure 5:**
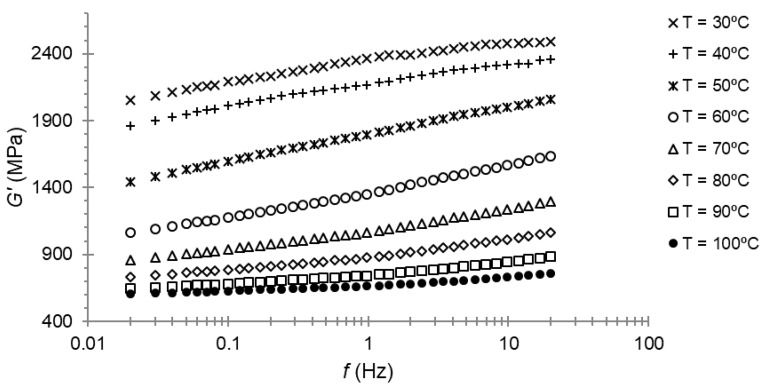
Storage modulus–frequency plot for PA66.

**Figure 6:**
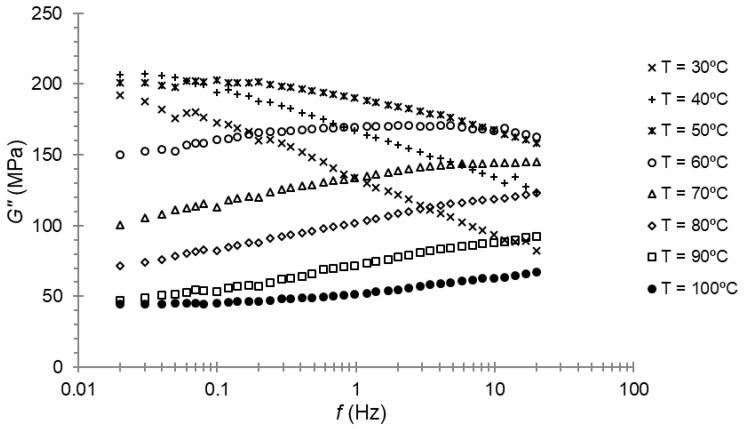
Loss modulus–frequency plot for PA66.

Frequency sweeps for the storage modulus and loss modulus for PA66 are presented in Figure 5 and Figure 6, respectively. Three repetitions of each test were performed, and we took the average of these results.

At first, the maximum of the loss modulus for 1 Hz of frequency was seen, as expected, at a temperature close to 50 °C; that is, the temperature at which the maximum of the loss modulus was found during the temperature sweep. When examining Figure 6, it can be seen that the maximum was reached between 40 and 50 °C for a lower frequency.

Once these frequency sweeps were performed, the experimental part of the method was finished.

The next step was to plot the flexural (or storage) modulus over the strain rate at the different temperatures tested. In this way, it was possible to find, for every temperature, the parameter *SE*_F_, as well as the flexural modulus at a reference strain rate of 1 mm/min.

**Figure 7:**
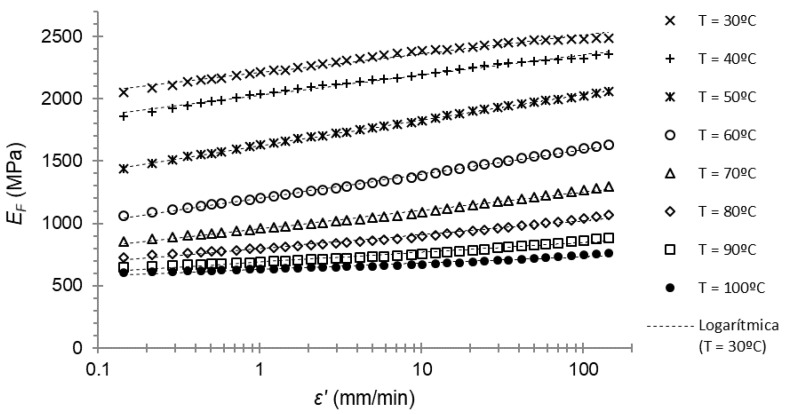
Flexural modulus–strain rate plot of the frequency sweep for PA66. Dotted lines are the mean-square-error fitting lines.

For PA66, the plot of the flexural modulus over the strain rate for every temperature is shown in Figure 7.

A logarithmic Equation (3) for every temperature was adjusted with a high coefficient of determination R^2^. The resulting slope of the equation was the parameter *SE*_F_ in (3). These results are shown in Table 1.

At first, the maximum of the loss modulus for 1 Hz of frequency was found, as expected, at a temperature close to 50 °C; that is, the temperature at which the maximum of the loss modulus was found at the temperature sweep. When examining Figure 6, it can be seen that the maximum was found between 40 and 50 °C for a lower frequency.

Moreover, the logarithmic tendency was confirmed again between the flexural modulus and the strain rate for this material (first hypothesis of this work).

### 3.2. Determination of the Shift Factor

In the frequency sweep of the DMA tests, every curve was determined at one specific temperature (Figure 5 and Figure 6). According the hypotheses, these curves could be horizontally shifted until they were superposed. This frequency–temperature superposition procedure was carried out by minimizing the squares-error between every couple of curves. The procedure resulted in just one curve with a larger frequency range.

As explained in Section 2.1.2, two cases were possible:

(a) If there was a maximum of the loss modulus over the frequency, two temperature subranges were necessary. On one hand, there was a first subrange that ranged from the minimum temperature to the temperature at which the maximum is found, adjusted with the Arrhenius equation. On the other hand, a second subrange that ranged from this last temperature to the maximum temperature, adjusted with the WLF equation.
(b) If there was no maximum of the loss modulus, just one range was applied, from the minimum to the maximum temperature. The choice of the shift function depended on the curve shape: if the loss modulus increased with temperature (a maximum was expected at a higher temperature), the Arrhenius equation was recommended; conversely, if the loss modulus decreased when the temperature increased, the WLF equation was recommended.

For PA66, two temperature subranges were defined: a first subrange from 30 to 50 °C, and a second one from 50 to 100 °C. The first subrange was adjusted with the Arrhenius Equation (7), and the second one with the WLF Equation (6). The frequency–temperature superposition returned the value of the shift coefficient $a_{T}$ for each temperature, defined as the relationship between frequencies:

(14) $$a_{T}=\frac{f_{T}}{f_{T_{\mathrm{ref}}}}$$

where $f_{T}$ and $f_{T_{\mathrm{ref}}}$ are the corresponding frequencies of the curves $T_{\mathrm{ref}}$. In the present case, *T*_ref_ was assumed to be 30 °C. For this reason, the shift factor at this specific temperature was 1. For the upper range, the shared value at 50 °C ensured the continuity of the shift coefficient.

The determination of the values of the shift coefficient was carried out by means of an algorithm of minimization of an objective function. A shift coefficient value was applied to the abscissae of each curve, calculated using the Arrhenius (from 30 to 50 °C) and WLF (from 50 °C) equations with the parameters undetermined. The objective function was calculated as the sum of squared errors between the junction points of the curve sections. This objective function was minimized, varying the parameters of the Arrhenius and WLF equations, using a nonlinear generalized reduced gradient algorithm. The Arrhenius Equation (7) gave:

(15) $$\log a_{T}=\frac{7388.73}{T}-24.39$$

where *T* is absolute temperature (K). In addition, the linearization of the WLF Equation (6) gave:

(16) $$\log a_{T}=\frac{-1}{0.17+\frac{9.91}{T-T_{\mathrm{ref}}}}$$

**Figure 8:**
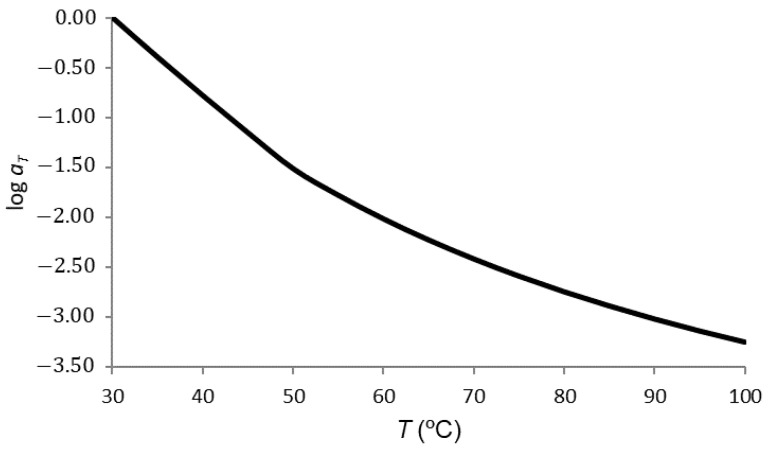
Shift factor versus temperature: a slight change can be seen in the glass-transition zone.

Figure 8 shows the results of these formulas.

**Figure 9:**
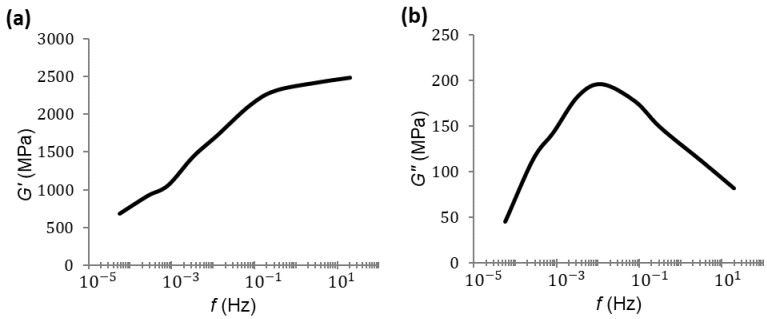
Master curve for the storage modulus (**a**) and loss modulus (**b**).

By applying the shift factor, the frequency range was extended. When applying this shift factor to the values of the frequencies at the different temperatures *T*, new shifted frequencies were obtained for *T*_ref_ = 30 °C (5): $f_{30}=a_{T}\cdot f_{T}$. Since the values of the shift coefficient were less than one, the points obtained at *T* > *T*_ref_ were shifted to the left (toward lower frequencies). By joining the curves thus displaced, the master curve could be drawn at *T*_ref_ (Figure 9).

### 3.3. Moduli Ratio

To calculate the moduli ratio, it was necessary to know the tensile modulus at least at one temperature. In this case, there were published data of tensile tests of PA66 at 23 °C [40]. The tensile modulus was calculated according to ISO 527-1/-2:

(17) $$E_{T}=\frac{\sigma_{0.0025}-\sigma_{0.0005}}{0.0025-0.0005}$$

The experimental value was $E_{T}\left(23\;℃,1\mathrm{mm}/\min\right)=2780\;\mathrm{MPa}$ for a strain rate of 1 mm/min. 

Due to the fact that the tensile modulus was known only at a given temperature (23 °C), the flexural modulus had to be shifted at 23 °C using the shift factor. As explained in Section 2.1.2, the shift factor had an influence on temperature and time or frequency due to the time–temperature superposition or frequency–temperature superposition, respectively. So, using the shift factor, a value of a mechanical property, such as the tensile modulus, measured at *T*_ref_, could be predicted at a different temperatures *T*.

Therefore, an extrapolation to 23 °C through mathematical formula (15) had to be applied in order to find this value. The extrapolation was considered acceptable, because shift functions were found in the temperature range of 30 to 100 °C. This extrapolation gave us a theoretically calculated (or “synthetic”) value of $E_{F}\left(23\;℃,1\mathrm{mm}/\min\right)$.

From an experimental value (at 30 °C and a 30 μm amplitude in a range of frequencies), the model could find a “synthetic” value (for 23 °C and 1.00 mm/min of strain rate). This was performed using the following procedure:

a. The known flexural modulus at 30 °C was the independent term (corresponding to 1 mm/min) of the adjustment of this modulus as a function of the strain rate (Table 1): $E_{F}\left(30\;℃,1\mathrm{mm}/\min\right)=2211\;\mathrm{MPa}$;
b. The frequency that corresponded to ${\dot{\varepsilon }}_{\mathrm{ref}}=1\;\mathrm{mm}/\min$ was calculated by applying (13): this gave $f_{\mathrm{ref}}=0.139\;\mathrm{Hz}$;
c. The shift factor at 23 °C $\left(a_{23}\right)$ was calculated (15) and then applied in (14) to calculate the corresponding frequency at that temperature: $f=a_{23}\cdot f_{\mathrm{ref}}=3.624\cdot 0.139=0.504\;\mathrm{Hz}$;
d. The flexural modulus $E_{F}\left(23\;℃\right)$ could be calculated on the logarithmic expression at $f=a_{23}\cdot f_{\mathrm{ref}}\;$: this was formula (3) taking the parameters of $T_{\mathrm{ref}}=30\;℃$, but shifting the strain rate to that corresponding to the shifted frequency; that is, $\dot{\varepsilon }=4\cdot 0.030\cdot 0.504\cdot 60=3.625\;\mathrm{mm}/\min$.

Then:

(18) $$E_{F}\left(23\;℃,1\mathrm{mm}/\min\right)=E_{F}\left(30\;℃,1\mathrm{mm}/\min\right)+SE_{F}\cdot \ln\left(3.625\right)$$

According to Table 1, $SE_{F}=65.0$. This resulted in a “synthetic” value of $E_{F}\left(23\;℃,1\mathrm{mm}/\min\right)=2295\;\mathrm{MPa}$.

Table 2 gives a summary of this procedure.

Finally, the moduli ratio was calculated as:

(19) $$MR=\frac{E_{T}\left(23\;℃,1\mathrm{mm}/\min\right)}{E_{F}\left(23\;℃,1\mathrm{mm}/\min\right)}=1.211$$

As a reminder, the units for strain rate are in mm/min in this work in order to compare the results with those found in tensile tests with a UTM, in which the strain rate was entered in mm/min, as well. 

### 3.4. Material Model: Temperature and Strain-Rate Dependence

In order to calculate the tensile modulus at every temperature, once the flexural modulus at every temperature and at 1 mm/min was measured, the tensile moduli at every temperature could be calculated by multiplying by moduli ratio:

(20) $$E_{T}\left(T,1\mathrm{mm}/\min\right)=MR\cdot E_{F}\left(T,1\mathrm{mm}/\min\right)$$

The first step was the calculation of the flexural modulus at one specific temperature for the reference strain ratio $E_{F}\left(T,{\dot{\varepsilon }}_{\mathrm{ref}}\right)$. In our case, ${\dot{\varepsilon }}_{\mathrm{ref}}=1\mathrm{mm}/\min$. This could be calculated through linear interpolation from experimental data of the storage modulus. However, in order to construct the general model from the DMA tests, we calculated the slope of the logarithmic adjustment of the flexural modulus as a function of the strain rate (*SE*_F_) at a reference temperature (in our case, at 30 °C).

Starting from this value, the flexural modulus (at ${\dot{\varepsilon }}_{\mathrm{ref}}$) for any other temperature could be calculated using the shift coefficient, as was done in the previous section.

(21) $$E_{F}\left(T,\;{\dot{\varepsilon }}_{\mathrm{ref}}\right)=E_{F}\left(T_{\mathrm{ref}}\;,\;a_{T}\cdot {\dot{\varepsilon }}_{\mathrm{ref}}\right)=E_{F}\left(T_{\mathrm{ref}},{\dot{\varepsilon }}_{\mathrm{ref}}\right)+SE_{F}\left(T_{\mathrm{ref}}\right)\cdot \ln\left(a_{T}\cdot {\dot{\varepsilon }}_{\mathrm{ref}}\right)$$

The tensile modulus at the reference conditions could be automatically calculated:

(22) $$E_{T}\left(T,{\dot{\varepsilon }}_{\mathrm{ref}}\right)=MR\cdot E_{F}\left(T_{\mathrm{ref}},a_{T}\cdot {\dot{\varepsilon }}_{\mathrm{ref}}\right)$$

Then, from (21) and (22), the general model (12) could be written for the material under study:

(23) $$E_{T}\left(T,\dot{\varepsilon }\right)=MR\cdot \left[E_{F}\left(T_{\mathrm{ref}}.{\dot{\varepsilon }}_{\mathrm{ref}}\right)+SE_{F}\left(T_{\mathrm{ref}}\right)\cdot \ln\left(a_{T}\cdot {\dot{\varepsilon }}_{\mathrm{ref}}\right)\right]+MR\cdot SE_{F}\left(T\right)\cdot \ln\dot{\varepsilon }$$

In the studied example, for PA66 at 23 °C, $E_{F}\left(30\;℃,1\mathrm{mm}/\min\right)$ was found to be 2211 MPa, $a_{T}=3.625$, *MR =* 1.211, and $SE_{F}\left(30\;℃\right)=65.0$. So, the model found:

(24) $$E_{T}\left(23℃,\dot{\varepsilon }\right)=1.211\cdot \left[2211+65\cdot \ln\left(3.625\cdot 1\right)+SE_{F}\left(T\right)\cdot \ln\left(\dot{\varepsilon }\right)\right]$$

with $\dot{\varepsilon }$ in mm/min. The value of the SE slope had to be obtained from the experimental curves (Table 1), taking the value of the closest temperature or interpolating. In the present case, we took $SE_{F}\left(23\;℃\right)\approx SE_{F}\left(30\;℃\right)=65.0$, and a more specific expression could be written:

(25) $$E_{T}\left(23\;℃,\dot{\varepsilon }\right)=2780+78.7\cdot \ln\dot{\varepsilon }$$

## 4. Validation

Some validations were conducted to close the loop. In order to do this, several values of the tensile modulus at different temperatures and strain rates, calculated using the presented method, were compared to experimental ones from the UTM tests.

### 4.1. Influence of Temperature

**Figure 10:**
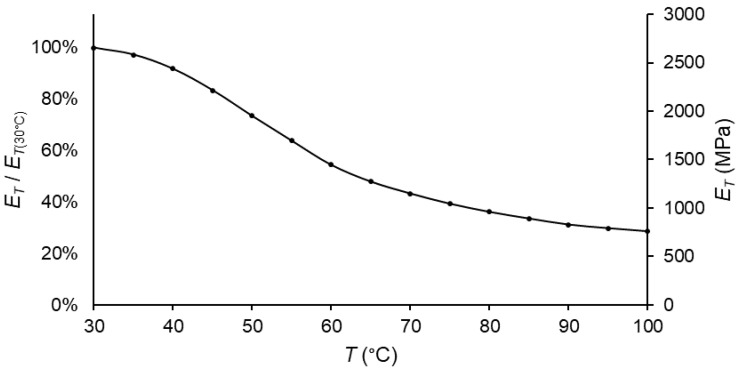
Tensile modulus vs. temperature plot for PA66, taking *T* = 30 °C as a reference.

Once *MR* was known, the tensile modulus could be calculated at every temperature (for $\dot{\varepsilon }=1\;\mathrm{mm}/\min)$ from the experimental data (Figure 7). The resulting plot of the tensile modulus over temperature for the material under study is shown in Figure 10.

As expected, the slope of the curve was higher if there was a subrange change; in other words, if there was a maximum of the loss modulus over the temperature. Moreover, in the same way as occurred for the storage/flexural moduli, the tensile modulus was lower at higher temperatures.

### 4.2. Validation by Temperature Dependence

In order to verify the values of the tensile modulus as a function of temperature, published stress–strain curves [40] at different temperatures for the same ZYTEL^®^ 103HSL material from DuPont^TM^ were compared to the prediction of the DMA results.

Taking the resulting values at 1 mm/min of the logarithmic fitting of the DMA results (Table 1) and applying the moduli ratio (1.211), this work assumed that it was possible to predict the tensile modulus. The resulting values were compared to the experimental ones for 40, 60, and 90 °C, as shown in Table 3.

As defined in the experimentation in DMA, the temperature range under study was from 30 to 100 °C. For this reason, the last temperature published, 120 °C, could not be compared, as it was out of range in our study.

The three hypotheses of this work were applied here: the logarithmic tendency of the moduli related to frequency, the equivalence between the flexural and tensile moduli (constant moduli ratio), and the equivalence between the frequency and strain rate. The error introduced by those assumptions remained under 5% for PA66.

In addition, these results were compared with those of the study by Mhanna et al. [2] of a PET–FRP laminate material. The decrease in the modulus in the elastic zone in both studies were of the same order.

### 4.3. Validation by Strain Rate Influence

Stress–strain curves at different strain rates for PA66 also were determined with a UTM at different strain rates, in order to verify the values given by the model of the tensile modulus (22, 23). Three repetitions were conducted for each strain rate. The resulting values of the tensile modulus are shown in Table 4.

The “synthesized” values of the tensile modulus were calculated by the model (25). Once we performed the experiment and the calculations, it was possible to compare both values for the different strain rates tested. For the PA66, the mathematical model for the tensile loop as a function of the strain rate also was well fitted: errors remained within the 5% range, as shown in Table 4.

## 5. Conclusions

As stated in the objectives of this work, the developed method, with the use of the mathematical model proposed in this paper, predicted tensile moduli of thermoplastic materials as a function of the temperature and strain rate from DMA data without using a UTM.

The use of the described development involved an important saving of time, as well as a significant decrease in the number of tests, when compared to the procedure with a UTM.

In order to verify the tensile moduli, a “tensile loop” was developed, and the following conclusions were drawn:

- The parameter defined as the moduli ratio presented a constant relationship between the tensile modulus and flexural modulus, with good results;
- Following the sinusoidal tendency of flexural tests, the equation between the strain rate, frequency, and amplitude was confirmed;
- There was a logarithmic tendency between the flexural modulus and frequency, as well as between the tensile modulus and strain rate;
- The divergence of the experimental results given by the tensile tests and the predicted values using this model as a function of these two variables was small (<5%) for PA66.

The method was applicable under the hypotheses of isotropy, homogeneity, small deformations, and linear viscoelasticity. Therefore, the estimated values could be used for a linear elastic behavior. This excluded the mechanical behavior under high stress or strain. In principle, this method could be applicable to other thermoplastic materials, under the stated hypotheses, as long as they are thermo-rheologically simple materials; that is, wherever the principle of time–temperature superposition is valid. Naturally, this should be validated in each case. 

Furthermore, the model was limited to the working range of the variables under study (strain rate and temperature).

Special attention must be paid to the glass-transition temperature. For the correct application of the model, it is necessary to determine this reference temperature. Significant variations occurred in the experimentation near this temperature. In the same way, the equation that related the shift factor with the temperature also was different below or above this temperature. Therefore, it is important to conduct an accurate experimental study around the glass-transition temperature, since important changes in the structural behavior of the material were observed experimentally, and there was a significant drop in the moduli. If that drop cannot be determined accurately, the model cannot be fed well enough to correctly estimate the tensile moduli. 

Finally, the method presented here, verified for a polyamide 66, must be tested with other thermoplastic materials to determine to what extent it can be applied reliably. In addition, a future work will expand the range of the variables (temperature and strain rate) in order to determine the limits of the method’s applicability.

## Figures and Tables

**Table 1:** Slope and independent terms of the logarithmic adjustment between flexural modulus and strain rate for PA66.

T (°C)	${\mathrm{SE}}_{F}\;\left(MPa\right)$	*E*_F_ (MPa) 1 mm/min	R^2^
30	65.0	2211	0.981
40	70.1	2029	0.992
50	88.7	1624	0.999
60	83.8	1203	0.997
70	63.6	957	0.992
80	48.0	799	0.982
90	33.7	690	0.974
100	21.6	632	0.963

**Table 2:** Flexural and tensile moduli at 23 °C for PA66.

T (°C)	A (mm)	$\dot{\varepsilon }$ (mm/min)	f (Hz)	*E*_F_ (MPa)	*E_T_* (MPa)
30	0.03	1.000	0.139	2211	-
30	0.03	3.625	0.504	2295	-
23	0.03	1.000	0.139	2295	2780

**Table 3:** Tensile modulus: experimental and predicted by the moduli ratio.

*T* (°C)	*E_T_* (MPa) Experimental	$E_{F}\;\left(T,1\;mm/min\right)\;\mathrm{Experimental}\;(\mathrm{MPa})$	$E_{T}=MR\cdot E_{F}\;(\mathrm{MPa})$	Error (%)
23	2780	2295	2780	0.00
40	2538	2029	2457	3.19
60	1450	1203	1457	0.48
90	846	690	836	1.18

**Table 4:** Tensile modulus by tensile test in UTM compared to “synthetic” tensile modulus of PA66 for different strain rates at 23 °C.

UTM Tests						Model	
Strain Rate (mm/min)	*E_T_* (MPa)			SD	*E_T_* (MPa)	*E_T_* (MPa)	Error (%)
	1	2	3				
1.000	2732	2795	2813	42	2780	2780	0.00
3.466	2777	2800	2871	49	2816	2878	2.20
12.011	2812	2885	2958	73	2885	2976	3.15
41.628	2838	3012	3036	108	2962	3073	3.75
144.270	3013	3072	3095	43	3060	3171	3.63
499.000	3045	3081	3222	93	3116	3269	4.91
